# Exploring a New Cueing Device in People Who Experience Freezing of Gait: Acceptance of a Study Design

**DOI:** 10.1155/2022/1631169

**Published:** 2022-12-07

**Authors:** Agnes Wilhelm, Tanja Riedl, Christian Paumann, Jessie Janssen

**Affiliations:** Institute of Therapeutic and Midwifery Sciences, Department of Health Sciences, IMC University of Applied Sciences Krems, Krems an der Donau, Austria

## Abstract

**Background:**

Freezing of Gait (FoG) is a disabling symptom of Parkinson's Disease (PD) and is defined as a “brief episodic absence or marked reduction of forward progression of the feet despite the intention to walk.” Compensatory strategies such as cueing and high frequency vibrotactile stimulation can reduce FoG severity and improve gait parameters. A new Sternal high frequency Vibrotactile Stimulation Device (SVSD) with cueing function has been developed, however the clinical effects of this device are yet to be fully investigated.

**Objective:**

The aim of this study was to investigate, if the proposed study design using a SVSD and gait analysis sensor insoles was acceptable for people with PD.

**Methods:**

This feasibility study was designed as a randomized cross-over study. Thirteen participants took part in a one off 60-minute data collection session. The acceptability of the study design was assessed with a mixed methods questionnaire considering each step of the study process. Secondary outcome measures were the feasibility of using the 10 Metre Walk Test (10MWT), the Freezing of Gait Score (FoG-Score), and Patient Global Impression of Change (PGI-C) with and without the SVSD.

**Results:**

The participants scored all aspects of the study design as very satisfactory. In addition, all participants could perform the secondary outcome measures and were deemed feasible. Feedback from open ended questions provided ideas and considerations for adaptations of future clinical studies.

**Conclusion:**

The proposed study design was acceptable for people with PD. *Implications*. This study design, with small adaptations, can be used for larger studies to evaluate the effect of an SVSD on FoG in people with PD.

## 1. Introduction

Parkinson's disease (PD) is a progressive neurodegenerative disease, which predominately occurs in the elderly population [1]. Freezing of Gait (FoG) is a disabling symptom of PD and manifests as a brief episodic absence or marked reduction of forward progression of the feet despite the intention to walk [2]. FoG reduces the patient's mobility, their independence, and has a significant impact on their quality of life [3]. Compensatory strategies such as cueing [4] and high frequency vibrotactile stimulation [5] can reduce FoG severity and improve gait parameters.

Cues are targets or references that support the execution of a movement [6]. External cues, such as auditory, visual, or sensory cues, are effective for improving gait parameters such as step length, speed, cadence, and stride length of PD patients [7]. A systematic review by Rocha et al. [7] found that different cues can have different effects on gait parameters. Auditory cues can increase the walking speed and decrease cadence, visual cues have a positive effect on walking speed and step length, and sensory cues can increase walking speed, stride length, and can decrease cadence [7]. A controlled study by Suputtitada et al. [8] compared the effects of visual, auditory, or somatosensory cueing on their own or in combination during walking in people with PD and found that walking speed and stride length improved significantly in all cues used independently. There was no additional benefit in combining different cues and it might be more important to use that cue that is the most practical for a specific situation [8].

In people with PD stride length, stride duration significantly changed from 2 seconds to 4 seconds prior to FoG [9]. Furthermore, Hausdorff et al. [10] showed that the variability of stride time during walking contributed to freezing. There is not enough evidence yet to determine that if a cueing device could influence these gait parameters and therefore have an effect on FoG [7].

The Sternal high frequency Vibrotactile Stimulation Device that was used in this study, was a wearable noninvasive focused stimulation device, attached to the sternum via a medical adhesive patch and used a quiet electric motor to produce high frequency vibrotactile stimulation. In 2021 a case report published by Tan et al. showed that a combination of cueing and a vibrotactile stimulation with a SVSD reduced FoG episodes and improved the mobility assessed with the Timed Up and Go test in two patients. However, evidence around this type of device is limited.

The aim of this study was to investigate if a study design using the above described SVSD and gait analysis sensor insoles together, while performing the FoG-Score and the 10 Metre Walk Test, was acceptable for participants. The objective was to gather information and the participants' feedback to use in further studies to investigate the effectiveness of the SVSD and to examine if the gait analysis sensor insoles could be used to evaluate an effect on FoG in people with PD.

## 2. Materials and Methods

### 2.1. Research Design

This study was conducted between January and April 2022 as a cross-sectional randomized cross-over study at a local University of applied sciences. The study was approved by the ethics committee of Lower Austria (GS4-EK-4/742-2021) and registered in the DRKS—German Clinical Trials Register (DRKS00028008).

### 2.2. Participants

Participants were recruited through seven PD self-support groups in Lower Austria. An e-mail with an information sheet was sent to the respective contact person of the PD self-support groups. A presentation with information about the study was given to individual PD groups. At the presentation the people had the opportunity to actively opt-in the study, or later via a telephone call.

People with PD, who were able to walk 2 × 10 m without assistance or assistive devices, had a shoe size between 36–47 (due to the gait analysis sensor insoles), and had a minimum score of 1 on the third question of the FoG Questionnaire (“Do you feel that your feet get glued to the floor while walking, making a turn or when trying to initiate walking (freezing)?” (0: never, 1: very rarely—about once a month, 2: rarely—about once a week, 3: often—about once a day, 4: always—whenever walking) were able to take part. To detect someone who is a definite or a probable freezer a question about “feeling glued” suffices [11].

When potential participants lacked the capacity to consent, they were excluded from the study. Exclusion criteria relating the SVSD included cardiac pacemaker, deep brain stimulation, sternal wounds, fractures, or skin conditions. Exclusion criteria relating to the gait analysis sensor insoles included participants over 135 kg body weight, wound or fractures on the feet, inability to wear closed shoes. All participants signed an informed consent form conforming with the Declaration of Helsinki.

### 2.3. Sternal high frequency Vibrotactile Stimulation Device

**Figure 1:**
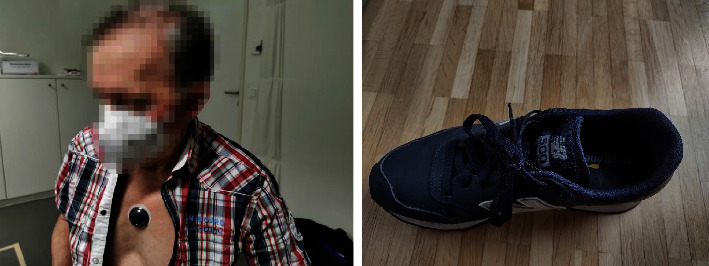
Application of the SVSD and gait analysis sensor insoles.

The SVSD was a noninvasive and wearable device for patients with PD that delivered rhythmic vibrotactile stimuli through a specialised frequency and pattern onto the sternum (Charco Neurotech Ltd.). It was certified as a class 1 medical product. The device measured 40 mm in diameter, 11 mm in height, and 17 g in weight, and was attached to the sternum with medical adhesive patches [12] (Figure 1). The SVSD was used with the default settings for example same vibration frequency for all participants, and no customisation of the stimulation settings was employed in this study.

### 2.4. Gait Analysis Sensor Insole

The gait analysis sensor insole was a wireless instrumented insole that recorded a range of movement data, displayed, and analysed it with the help of a software program (StaPPtronics GmbH) (Figure 1). It was a class 1 medical product certified in accordance with Directive 93/42/EEC (Medical Device Directive). Each insole had 12 textile pressure sensors that recorded the pressure distribution while walking. In addition, each insole had an inertial measuring unit (IMU), which recorded data using an acceleration sensor. Symmetry of the gait pattern and important gait parameters such as step length, cadence, or step duration were measured with this device. There was no conflict of interest with either Charco Neurotech Ltd. or StaPPtronics GmbH other than technical support.

### 2.5. Procedure

After signing the informed consent, the two devices were set in place. The SVSD was applied on the sternum, the gait analysis sensor insoles were put into the participants shoes and connected to the gait analysis software.

Demographic data such as age, gender, duration of PD, confounders including diseases of the central or peripheral nervous system, and the occurrence of acute pain while walking and the point of time when the last PD medication was taken, were collected. The testing order was randomised for vibration and FoG-Score and the 10 Metre Walk Test (10MWT) via Research Randomizer (https://www.randomizer.org/). Four different orders were used (Table 1).

Regardless of the randomization order, every participant completed a pilot FoG-Score [13]. The FoG-score included standing up from a chair, initiating walking, walking for one metre, followed by a 360° clockwise rotation, and a 360° anticlockwise rotation, walking two metres and finally going through a door. Rotations of 360° were considered the most effective trigger for FoG. The FoG-Score was conducted three times. The first time without any dual tasks, the second time whilst holding a glass of water, the third time counting backward 100–7 while holding a glass of water [11]. The evaluation of the FoG is based on the phenomenology of the leg movements [13]. The leg movements are defined into 3 categories: festination, trembling in place, and no movement/akinesia. The assessment was conducted by two experienced neurological physiotherapists without any technical equipment. The minimal clinically relevant change (MCRC) for improvement is three scale points [14].

The 10MWT was used to reliably measure gait speed of individuals with PD in fast conditions [15]. The task was to walk as fast as they can safely and stop after the 10-metre mark. The participants had a dynamic start and no pilot trial [16]. The minimally detectable change (MCD) in people with PD is 0.25 m/s [17] and the minimally clinically important difference (MCID) in the geriatric population for a substantial change is 0.13 m/s [18]. The normative value in fast conditions in the population aged from 60 to 69 years the average walking speed is 1.93 m/s for men and 1.77 m/s for women [19].

Both assessments were completed one time with the SVSD turned on and one time turned off, depending on the randomization order. After the FoG-Score and 10MWT, the participants scored a Visual Analogue Scale (FoG-VAS and 10MWT-VAS) regarding their personal satisfaction with the test and had a 3-minute break.

After the completion of both assessments, the participants' acceptance of the study design was assessed via a questionnaire about their satisfaction with the information they received about this study, the overall time frame, the test sequence, the FoG-Score, the 10MWT with and without the SVSD, and the overall study process, which would answer the primary research question of the study (Table 2). Each question consisted of a 5-point Likert scale ranging from 1 (very satisfactory) to 5 (very unsatisfactory). The participants also had the opportunity to comment on any of the aspects of the study and put in suggestions and feedback in open questions. Three open-ended questions were asked to the participants: “What would be important for participation in another (follow-up) study from your point of view?,” “Is there anything else you think we could improve?,” and “Is there anything else you would like to tell us about the study in general?.”

The subjective difference in completing the 10MWT and the FoG-Score with and without the SVSD was compared and assessed with the Patient Global Impression Scale for Change, a Patient Reported Outcome (PRO) instrument, which used a 7-point Likert scale (1 = very much better with SVSD, 4 = no difference, 7 = very much worse with SVSD) [20]. The scores 1–3 of the PGI-C could be considered clinically meaningful improvements [21]. The subjective “much improved” and “very much improved” ratings can indicate moderately important and substantial improvement [22].

### 2.6. Data Processing and Analysis

All quantitative data were documented in SPSS (IBM SPPSS Statistics version 28.0.1.0), checked for accuracy, and missing data were coded. Data were checked for normal distribution with the Shapiro-Wilcoxon test. Most data were nonparametric therefore all data were displayed in median and interquartile range (IQR). No inferential statistical analysis was conducted due to the small sample size.

All qualitative data were recorded on an Excel document. Using a deductive content analysis approach all comments were coded to the following three categories: (1) overall study process, (2) SVSD, (3) gait analysis sensor insoles. One researcher (AW) coded the comments to the categories and one researcher (JJ) checked a random sample for accuracy.

## 3. Results

**Figure 2:**
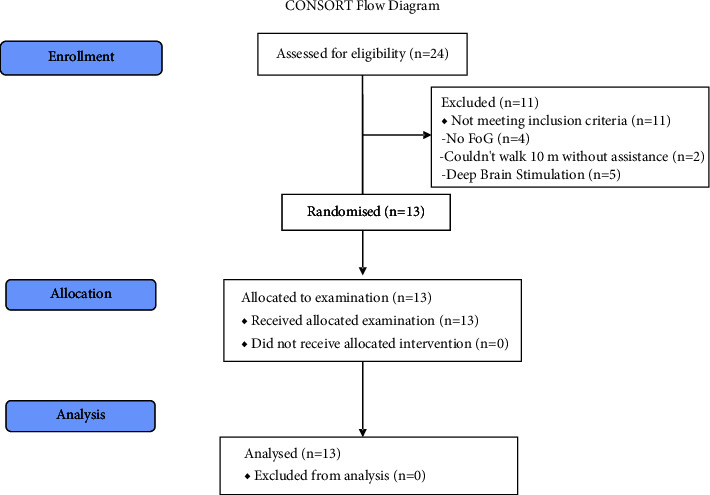
CONSORT flow diagram on enrolment, allocation, and analysis.

Thirteen people with PD, nine men and four women with a Hoehn and Yahr score ranging between 1 and 4 participated in the study. The flowchart for the Consolidated Standards of Reporting Trials (CONSORT) is shown in Figure 2. Median age was 66 years old (IQR 63 to 71) with a median duration of PD diagnosis of 6 years (IQR 4 to 8). Out of the thirteen participants, three reported pain during walking, while two people listed other diagnoses of the central nervous system. Most participants, seven out of 13, reported medium to strong limitations due to their PD diagnosis. The median last intake of PD medication was 1 hour (IQR 0.5 to 1.5 hours) prior to study start.

### 3.1. Acceptability of Study Design

In Table 2 the results are listed for the primary research question: How acceptable is this study design for people with PD? In all nine questions the participants noted that they were very satisfied with the components of the study design.

### 3.2. Feasibility of Using Secondary Outcome Measures

The secondary research questions focused on the feasibility of using the FoG-Score, 10MWT, and PGI-C questionnaire in the study. Both the FoG-Score and the 10MWT were completed by all the participants in the study and was therefore concluded to be feasible (Table 3). A higher range in FoG-Score occurred when the SVSD was switched off (mean 1, IQR 0–8.5) than with the device switched on (mean 0, IQR 0–5.5). When the SVSD was switched on 5/13 participants experienced FoG and 7/13 when the SVSD was switched off. Most of these participants (5/5 and 5/7, respectively) experienced FoG during the turning clockwise and counter clockwise while carrying a glass of water and counting backwards from 100–7. One participant experienced FoG while starting to walk and two participants while walking through the door.

During the 10MWT none of the participants experienced FoG, however from all, except one participant due to technical issues, gait parameters could be recorded.

In the last part of the study the participants were asked in the PGI-C form if they experienced a difference with and without the SVSD switched on during the FoG-Score and 10MWT. All participants could answer these two questions and listed that no difference was observed (FoG-Score 4 (IQR 3-4), 10MWT 4 (IQR 3-4)).

The answers to the open-ended questions could be summarised into three categories. First regarding the study process in general, second regarding the SVSD, and third regarding the gait analysis sensor insoles (Table 4).

## 4. Discussion

The aim of this study was to evaluate the acceptability of this study design for people with PD, not to assess the efficacy of the used device. The 13 participants found that the information that the participants received, the time frame, and all 4 randomisation orders for the test sequences for this study were very acceptable for people with PD. Also, the FoG-Score and the 10MWT were feasible while wearing the gait analysis sensor insoles and having the SVSD switched off or on during the testing. The gait analysis sensor insoles collected useful data to calculate double step length (cm), cadence, and symmetry (% right leg) while performing the 10MWT. This study design could be used for a bigger study with more participants to evaluate the effect of the SVSD on FoG in people with PD.

In the study conducted by Snijders et al. [11], 24 of the 32 people (75%) who reported subjective FoG with the FoG-questionnaire (“feet being glued to the floor”) experienced FoG during the clinical assessment. In our study a relatively lower number of participants experienced FoG during the FoG-Score: 8/13 (61.5%) when the SVSD was turned on and 6/13 (46.1%) when the SVSD was turned off. In accordance with the study by Snijders et al. [11], a 360° turn and additional dual tasks was the most effective test to provoke FoG. In our study turning clockwise and counter clockwise and carrying a glass of water while doing the calculation 100–7 backwards had the highest FoG rate with 5 out of the 5 participants who froze (100%) with the SVSD turned on and 5 out of the 7 participants who froze (71.4%) with the SVSD switched off.

The 10MWT is recommended as an assessment of walking speed in people with PD and can be used to identify changes [16]. This study showed that the 10MWT can be performed by people with PD while wearing gait analysis sensor insoles and a SVSD. We used a dynamic start and no pilot trial, which is in accordance with the study by Lindholm et al. [16]; which identified no differences in dynamic or static starting conditions or one versus two trials in people with PD. The study by Lindholm also found that 63 of 151 participants (42%) experienced FoG, but none during the 10MWT. In our study also, none of the 13 participants experienced FoG while performing the 10MWT under fast conditions. The walking speed of the participants in our study was calculated by dividing 10 metres by the time taken to complete 10MWT and was 1.4 m/s without the SVSD and 1.6 m/s with the SVSD switched on. This is slightly slower compared to the normative data with healthy adults in fast condition aged 60 to 69 years, which is 1.7–1.9 m/s [19]. According to Perera et al. [18] a 0.10 m/s is a substantial change for gait speed in older adults but Steffen and Seney [17] found that 0.25 m/s is the minimal detectable change (MDC) in people with PD. The study by Lindholm found a mean walking speed with 0.9 m/s in 63 people with PD and a history of FoG. Compared to that study our participants had a higher walking speed. Age (mean 68 years versus mean 67 years in our study) and duration of PD (mean 4 years versus mean 6 years in our study) were similar to Lindholm's participants. Due to our recruitment process in self-support groups and the need for the ability to come to the testing site on their own we might have had higher functioning participants, compared to the participants in the Lindholm study, who were recruited in a university hospital neurology outpatient clinic. We also excluded any walking aids due to Item #8 and #12 in the FoG-Score where the participants should carry a glass of water and open a door. In the Lindholm study 13% used a walking aid during the test. The use of a walking aid can have an influence on the ability to detect changes in walking speed [23].

The feedback gathered from the participants about the study process in the open questions can be used for further studies. The feedback was overall positive and showed an interest in participating in another study. One important feedback that could also influence the outcome of the assessments is the time of day of testing and the last intake of PD medication [24–26]. Our participants had a median last intake of their PD medication 1 hour ago. The most common PD medication is Levodopa. The drug concentration when taken orally for levodopa is highest between 0.5 and 2 hours after intake [27], which indicated that our participants were in their On-Phase during the testing. There were no detailed questions about what kind of medication they took in our study.

Another feedback pointed out that there was a pilot run for the FoG-Score and no pilot run for the 10MWT. As the study by Lindholm et al. [16] shows, there is no significant difference between one or two trials in the outcome for the walking speed, so a pilot run has no influence on the outcome of the 10MWT.

Several participants wished for more information about the devices and the underlying mechanisms. Prior to the study we supplied limited information about the SVSD to the participants, to limit the influence on the participants. However, some participants researched the device before participation, others had a different idea altogether about what the device could do and thought it was “some kind of alarm device.” Therefore, the participants had different information about the function of the SVSD before the testing started. The perception that the device could help to improve walking, could have had a placebo effect and influence the outcome of the assessments from the participants' point of view [28, 29]. In addition, the researchers could have been affected by a biased view. Although two researchers rated the FoG-Score, it would be better to have blinded assessors in the next study.

The feedback for the SVSD was positive and there were no comments about any objectionable experiences. Usage of the device in everyday life could be imagined. Some people wished for a stronger vibration and an individual setting of the frequency. All participants had the default setting, and were not given access to the customising feature so no changes were made to tailor the stimulation, as the study was aimed to evaluate the overall acceptability of this study design and not the efficacy. However, in further studies a personalised frequency could enhance the study design.

### 4.1. Limitations

Although this study design proved to be acceptable and feasible there were some limitations and improvements to be considered for another study. These results are limited by a relatively small numbers of individuals with 13 participants. The people in this study had reduced occurrence of FoG and increased walking speed in the 10MWT compared to similar studies [11, 16]. 46–61% of the participants did not freeze during the FoG-Score, although they subjectively reported FoG before. Including other activities in the testing of FoG could be advisable. One participant pointed out that climbing the stairs was an individual FoG trigger, also the turning and barrier course mimic real-life environments that elicit FoG [30]. And Snijders et al. [11] found that a rapid 360° turn was more effective in provoking FoG than with a normal speed. The effect of the SVSD on FoG needs to be determined in further studies with more participants. Considering one participant's feedback that wished for a longer wearing duration, the 6 minute Walking Test (6MWT) could be used [31]. Our population had an average walking speed of 1.4–1.6 m/s compared to Lindholm with 0.9 m/s. Our findings may, therefore, not be applicable to people with PD with a slower walking speed. Additionally, we should consider the possibility of the placebo effect within the participants and the researchers, so there should be some information prepared that everybody involved gets beforehand to minimise this effect. Patients and public involvement (PPI) can help put the information in nonacademic language to ensure that everybody has the same level of knowledge.

## 5. Conclusions

This study design proved to be very acceptable for people with PD. The use of both the FoG-Score and the 10MWT were feasible while additionally wearing gait analysis sensor insoles and a SVSD. The gait analysis sensor insoles can gather valid data for gait parameters during both assessments. Further studies, specifically designed to explore the efficacy of the SVSD, should be designed on a larger scale and consider medication intake, training runs of all assessments, tailored information about the function of the device and personalised frequency of the SVSD.

## Figures and Tables

**Table 1:** Randomization order, (+) = with SVSD, (−) = without SVSD.

Order 1 (*n* = 3)	Order 2 (*n* = 3)	Order 3 (*n* = 4)	Order 4 (*n* = 3)
FoG-score (+)	10MWT (+)	FoG-score (−)	10MWT (−)
10MWT (+)	FoG-score (+)	10MWT (−)	FoG-score (−)
FoG-score (−)	10MWT (−)	FoG-score (+)	10MWT (+)
10MWT (−)	FoG-score (−)	10MWT (+)	FoG -core (+)

**Table 2:** Overview of the questions and answers to the acceptability of the study design. Scale 1 (very satisfied)–5 (very unsatisfied), FoG-score = freezing of gait score, and 10MWT = 10 metre walk test.

		Median	IQR
1	How satisfied were you with the information you received before participating in the study?	1 (very satisfied)	1-2
2	How well did the information in the consent form prepare you for what to expect in the study?	1 (very good)	1-2
3	How satisfied were you with the overall time frame of the study process?	1 (very satisfied)	1-1
4	How satisfied were you with the test sequence?	1 (very satisfied)	1-1
5	How satisfied were you with the FoG-score without the sternal vibrotactile stimulation device?	1 (very satisfied)	1-2
6	How satisfied were you with the FoG-score with the sternal vibrotactile stimulation device?	1 (very satisfied)	1-2
7	How satisfied were you with the 10MWT test without the sternal vibrotactile stimulation device?	1 (very satisfied)	1-2
8	How satisfied were you with the 10MWT test with the sternal vibrotactile stimulation device?	1 (very satisfied)	1-2
9	How satisfied were you with the study process overall?	1 (very satisfied)	1-1

**Table 3:** Results of the secondary outcome measures FoG-score and 10MWT. ^*∗*^one participant is missing *n* = 12.

*N* = 13	Without SVSD Median (IQR)	With SVSD Median (IQR)
FoG-score	1 (0–8.5)	0 (0–5.5)
FoG-VAS	4.4 (2.6–6.3)	5.1 (3.0–6.6)
10MWT (seconds)	7.0 (5.57–8.08)	6.4 (5.68–8.04)
10MWT (m/s)	1.4 (1.24–1.8)	1.6 (1.24–1.76)
10MWT-VAS	8.4 (5.75–9.40)	7.7 (5.25–9.3)
Double step length (cm)	155 (127–167)^*∗*^	161 (118–178)^*∗*^
Cadence	121 (117–133)^*∗*^	120 (115–133)
Symmetry (% right leg)	50 (48–51)^*∗*^	50 (49–52)

**Table 4:** Feedback from the open questions about the study.

Overall study process	SVSD	Gait analysis sensor insoles
Interest in participating in another study	SVSD pleasant and well noticeable	Insoles are comfortable
Time of day of testing and taking medication (on- and off-phase)	Vibration could be stronger	Insoles soles don't change gait
Pilot run for the 10 MWT	Individual setting of frequency would be good	
Stair climbing could be an individual trigger for FoG	Could imagine use in everyday life	
More information on mechanism of action prior to taking part	Longer wearing time to say more about the device	
Thought that the device was “some sort of alarm”	Quick habituation effect, vibration faded out, did not experience SVSD as cueing	

## Data Availability

The quantitative data used to support the findings of this study have been deposited in the GitHub repository (https://doi.org/10.5281/zenodo.7092731).
